# Independent Associations of Appendicular Skeletal Muscle Mass Index and Waist Circumference with Site-Specific Bone Mineral Density in Postmenopausal Women: A Real-World Cross-Sectional Study

**DOI:** 10.3390/healthcare14162663

**Published:** 2026-08-21

**Authors:** Pattama Tongdee, Sajeera Kupittayanant, Khanidtha Marungrueng, Sayah Ongsricharoenbhorn, Porntip Nimkuntod

**Affiliations:** 1 School of Obstetrics and Gynecology, Institute of Medicine, Suranaree University of Technology, Nakhon Ratchasima 30000, Thailand; 2 School of Preclinical Sciences, Institute of Science, Suranaree University of Technology, Nakhon Ratchasima 30000, Thailand; sajeera@sut.ac.th; 3 School of Occupational Health and Safety, Institute of Public Health, Suranaree University of Technology, Nakhon Ratchasima 30000, Thailand; mkhanidtha@sut.ac.th; 4 Suranaree University of Technology Hospital, Nakhon Ratchasima 30000, Thailand; sayah.on@g.sut.ac.th; 5 School of Internal Medicine, Institute of Medicine, Suranaree University of Technology, Nakhon Ratchasima 30000, Thailand

**Keywords:** bone mineral density, appendicular skeletal muscle mass index, waist circumference, postmenopausal women, body composition, osteoporosis

## Abstract

**Highlights:**

**What are the main findings?**

- Higher appendicular skeletal muscle mass index was independently associated with higher bone mineral density at the lumbar spine, total hip, and femoral neck.
- Waist circumference remained independently associated with bone mineral density at the total hip, but not at the lumbar spine or femoral neck, after multivariable adjustment.

**What are the implications of the main findings?**

- Assessment of muscle mass provides additional information beyond body weight or body mass index when evaluating skeletal health in postmenopausal women.
- Combining routine waist circumference measurement with bioelectrical impedance analysis-derived muscle assessment may provide complementary information on body composition and skeletal health in outpatient practice.

**Abstract:**

**Background/Objectives**: Postmenopausal aging is accompanied by progressive loss of skeletal muscle mass and increased abdominal adiposity, both of which may influence bone health through distinct mechanical and metabolic pathways. However, their independent associations with bone mineral density (BMD) at different skeletal sites remain incompletely characterized. This study investigated the independent associations of appendicular skeletal muscle mass index (ASMI) and waist circumference (WC) with site-specific BMD in postmenopausal women. **Methods**: This cross-sectional study included 345 postmenopausal women who attended a university hospital menopause clinic in Thailand. Body composition was assessed using multi-frequency bioelectrical impedance analysis, and ASMI was calculated according to the Asian Working Group for Sarcopenia (AWGS) 2019 consensus criteria. WC was measured using standardized anthropometric procedures. BMD at the lumbar spine (L1–L4), total hip, and femoral neck was determined by dual-energy X-ray absorptiometry. Multivariable linear regression analyses were performed to evaluate the independent associations of ASMI and WC with site-specific BMD after adjustment for age, years since menopause, hormone replacement therapy use, osteoporosis medication use, calcium supplementation, vitamin D supplementation, and fracture history. Robust standard errors were used in the final models. **Results**: Higher ASMI was independently associated with higher BMD at the lumbar spine (standardized β = 0.362, *p* < 0.001), total hip (standardized β = 0.371, *p* < 0.001), and femoral neck (standardized β = 0.353, *p* < 0.001). WC was positively associated with total hip BMD (standardized β = 0.158, *p* = 0.006), whereas no statistically significant associations were observed at the lumbar spine (standardized β = 0.009, *p* = 0.899) or femoral neck (standardized β = 0.105, *p* = 0.092). **Conclusions**: ASMI was consistently associated with BMD across all measured skeletal sites, whereas the association between WC and BMD varied according to skeletal region. These findings support the complementary assessment of skeletal muscle mass and central adiposity for characterizing body composition and skeletal health in postmenopausal women.

## 1. Introduction

Osteoporosis is one of the most common chronic musculoskeletal disorders affecting older adults and represents a major public health challenge worldwide. Progressive loss of bone mineral density (BMD) and deterioration of bone microarchitecture substantially increase the risk of fragility fractures, disability, loss of independence, and premature mortality, particularly among postmenopausal women [1,2,3,4]. Owing to the rapid aging of populations globally, identifying modifiable factors associated with skeletal health has become increasingly important for fracture prevention and healthy aging.

Menopause is accompanied by profound endocrine and metabolic changes that affect not only bone but also body composition. Declining estrogen concentrations accelerate bone remodeling while simultaneously promoting loss of skeletal muscle mass and redistribution of adipose tissue toward the abdominal region [2,5,6,7]. Consequently, postmenopausal women frequently experience concurrent reductions in muscle mass together with increasing central obesity. These body composition changes have attracted considerable clinical interest because skeletal muscle and adipose tissue interact with bone through multiple biomechanical, endocrine, and inflammatory pathways.

Traditionally, greater body weight or body mass index (BMI) has been considered beneficial for bone health because of increased mechanical loading on the skeleton. However, BMI cannot differentiate lean tissue from adipose tissue and therefore provides limited information regarding the biological components that may differentially influence bone metabolism. Recent studies suggest that appendicular skeletal muscle mass is positively associated with BMD, whereas the relationship between abdominal adiposity and skeletal health remains inconsistent. Previous studies have also reported associations between skeletal muscle or lean mass and BMD, including studies in postmenopausal women. While increased body weight may enhance mechanical loading, excess central adiposity has also been linked to chronic low-grade inflammation, altered adipokine secretion, and adverse metabolic effects that may negatively influence bone remodeling. These conflicting findings suggest that individual body composition components should be evaluated separately rather than relying solely on BMI [8,9,10,11,12,13,14].

The relationship between body composition and bone health may also vary according to skeletal site. Trabecular bone, which predominates in the lumbar spine, is metabolically more active and responds rapidly to hormonal and inflammatory stimuli, whereas cortical-rich regions such as the hip are influenced more strongly by long-term mechanical loading. Despite these biological differences, relatively few clinical studies have simultaneously evaluated appendicular muscle mass and abdominal adiposity as independent correlates of BMD at multiple skeletal sites using measurements that are readily applicable in routine clinical practice [15,16,17,18,19,20].

Bioelectrical impedance analysis (BIA) and waist circumference (WC) are inexpensive, non-invasive, and widely available tools that can be incorporated into routine outpatient assessment without substantial additional cost or patient burden. Clarifying how these routinely obtainable measures relate to site-specific BMD may improve clinical assessment of skeletal health beyond conventional anthropometric indices [21,22,23,24,25,26,27]. Therefore, the present study aimed to investigate the independent associations between appendicular skeletal muscle mass index (ASMI), WC, and site-specific BMD at the lumbar spine, total hip, and femoral neck in postmenopausal women attending a routine outpatient menopause clinic.

We hypothesized that appendicular skeletal muscle mass would show consistent positive associations with BMD across skeletal sites, whereas the association between WC and BMD would be site-specific.

## 2. Materials and Methods

### 2.1. Study Design and Setting

This cross-sectional study was conducted at the Menopause Clinic, Suranaree University of Technology Hospital, Nakhon Ratchasima, Thailand, between January 2025 and June 2026. The study aimed to investigate the independent associations of appendicular skeletal muscle mass and WC with site-specific BMD among postmenopausal women receiving routine outpatient care.

The study was designed and reported in accordance with the Strengthening the Reporting of Observational Studies in Epidemiology (STROBE) statement [21].

### 2.2. Ethical Approval

The study protocol was approved by the Human Research Ethics Committee of Suranaree University of Technology (Approval No. 200/2567). All eligible participants received a participant information sheet and a written informed consent form. Written informed consent was obtained from all participants before enrollment and prior to any study procedures. The study was conducted in accordance with the ethical principles of the Declaration of Helsinki [22].

### 2.3. Participants

Women attending the menopause clinic who had undergone dual-energy X-ray absorptiometry (DXA) as part of routine clinical care were consecutively invited to participate. Eligible participants were postmenopausal women with at least 12 months of spontaneous amenorrhea. Menopausal status was determined from clinical history without routine hormonal confirmation [5].

Participants were excluded if they had conditions that could substantially affect body composition assessment or bone metabolism, including inability to stand independently during BIA, implanted cardiac pacemakers or cardioverter-defibrillators, secondary osteoporosis (e.g., primary hyperparathyroidism or Paget’s disease), chronic systemic corticosteroid therapy (>3 months), or end-stage kidney disease requiring dialysis. A total of 346 eligible postmenopausal women were enrolled during the study period. One participant subsequently withdrew from the study; therefore, 345 participants completed the study assessments and were included in the final analysis. The number of women screened before enrollment and those who declined participation were not systematically recorded.

### 2.4. Anthropometric Measurements

Body weight and height were measured using calibrated instruments with participants wearing light clothing and no shoes. BMI was calculated as weight (kg) divided by height squared (m^2^). WC was measured at the midpoint between the lower rib margin and the iliac crest at the end of normal expiration using a non-stretch measuring tape. WC was used as the primary anthropometric measure of central adiposity. WC was selected for the primary analyses because it is a simple, routinely obtainable measure with direct applicability to outpatient clinical practice.

### 2.5. Body Composition Assessment

Body composition was assessed using a multi-frequency BIA (Charder MA601, Charder Electronic Co., Ltd., Taichung, Taiwan). Measurements were performed as part of routine outpatient assessment. To minimize variability related to bladder volume, participants were asked to empty their bladder before measurement. Appendicular skeletal muscle mass (ASM) was calculated as the sum of lean mass in both arms and both legs, and ASMI was calculated as ASM divided by height squared (kg/m^2^). Low muscle mass was defined according to the Asian Working Group for Sarcopenia (AWGS) 2019 criterion for women (ASMI < 5.7 kg/m^2^) [23]. Total fat mass, percentage body fat, and trunk fat mass were also obtained from the BIA assessment. Other premeasurement conditions were not strictly standardized because BIA was performed as part of routine outpatient assessment.

### 2.6. Bone Mineral Density Assessment

BMD was measured at the lumbar spine (L1–L4), total hip, and femoral neck using DXA (Lunar Prodigy, GE Healthcare, Chicago, IL, USA). Daily quality assurance procedures were performed according to the manufacturer’s recommendations. Absolute areal BMD was recorded in g/cm^2^ at each skeletal site.

Bone status was classified according to the World Health Organization (WHO) criteria as normal (T-score ≥ −1.0), osteopenia (T-score between −1.0 and −2.5), or osteoporosis (T-score ≤ −2.5) [28,29]. T-scores were used for the clinical classification of bone status, whereas absolute BMD (g/cm^2^) was used as the continuous outcome in the correlation and multivariable linear regression analyses.

### 2.7. Sample Size Estimation

The sample size was determined *a priori* according to the approved study protocol. The original study was designed to investigate the associations between body composition and bone health, including comparisons of BMD between postmenopausal women with low and normal ASMI.

Sample size estimation was performed using Stata Statistical Software, Release 18 (StataCorp LLC, College Station, TX, USA) based on a comparison between two independent groups. Assuming a two-sided significance level (α) of 0.05 and a statistical power of 80%, the minimum required sample size was estimated to be 195 participants per group (390 participants in total). To compensate for an anticipated 10% rate of incomplete or missing data, the planned recruitment target was increased to 430 participants.

Consecutive sampling was applied throughout the ethics-approved recruitment period. A total of 345 participants completed the study assessments and were included in the final analyses. Although the planned recruitment target was not fully achieved, the available sample provided a substantial number of observations relative to the number of covariates included in the multivariable regression models [30,31].

### 2.8. Statistical Analysis

Statistical analyses were performed using Stata Statistical Software, Release 18 (StataCorp LLC, College Station, TX, USA). A two-sided *p*-value < 0.05 was considered statistically significant.

Continuous variables were assessed for normality using the Shapiro–Wilk test. Normally distributed variables are presented as mean ± standard deviation (SD), whereas non-normally distributed variables are presented as median and interquartile range (IQR). Categorical variables are presented as frequencies and percentages.

Baseline characteristics were compared using the independent-samples *t* test or the Mann–Whitney *U* test for continuous variables, as appropriate, and the chi-square test or Fisher’s exact test for categorical variables.

Pearson’s correlation analysis was performed to evaluate the unadjusted relationships between demographic, anthropometric, and body-composition variables and absolute BMD (g/cm^2^) at the lumbar spine, total hip, and femoral neck. Multivariable linear regression analyses were subsequently performed separately for absolute BMD at each skeletal site. ASMI and WC were the primary body-composition variables of interest. The primary models included age and years since menopause as *a priori* clinically relevant covariates. Expanded models additionally included available bone-related clinical factors, including hormone replacement therapy (HRT) use, osteoporosis medication use, calcium supplementation, vitamin D supplementation, and fracture history.

Model assumptions were evaluated by examining linearity, residual distributions, homoscedasticity, multicollinearity, and potentially influential observations. Multicollinearity was assessed using variance inflation factors (VIFs), and heteroskedasticity was assessed using the Breusch–Pagan/Cook–Weisberg test. Residual and influence diagnostics, including studentized residuals and Cook’s distance, were also examined. Because mild heteroskedasticity was detected in the lumbar spine model, heteroskedasticity-robust standard errors were applied consistently across the final multivariable models. No observation was considered sufficiently influential to warrant exclusion from the final analyses. Regression results are reported as unstandardized regression coefficients (β), 95% confidence intervals (CIs), standardized regression coefficients (standardized β), and corresponding *p* values. Site-specific T-scores were additionally examined as supplementary continuous outcomes using the same multivariable modeling approach.

Multivariable analyses were conducted using complete cases for the variables included in each model; therefore, the analytic sample size varied slightly across skeletal sites.

## 3. Results

### 3.1. Baseline Characteristics of the Study Population

A total of 345 postmenopausal women were included in the analysis. Based on the AWGS 2019 cutoff for women, 177 participants (51.3%) were classified as having low ASMI (<5.7 kg/m^2^), whereas 168 (48.7%) had normal ASMI. The mean age of the overall study population was 60.6 ± 8.2 years.

Age and years since menopause did not differ significantly between the normal- and low-ASMI groups (both *p* > 0.05). Compared with women with low ASMI, those with normal ASMI had significantly greater body weight, height, BMI, WC, total fat mass, percentage body fat, and trunk fat mass (all *p* < 0.001). Mean ASMI was 6.34 ± 0.50 kg/m^2^ in the normal-ASMI group and 5.14 ± 0.40 kg/m^2^ in the low-ASMI group.

Hormone replacement therapy (HRT) use and fracture history did not differ significantly between groups. Osteoporosis medication use was more frequent among women with low ASMI than among those with normal ASMI (21.0% vs. 6.5%, *p* < 0.001). Calcium and vitamin D supplementation did not differ significantly between groups. The number of available observations varied for some variables because of missing data (Table 1).

### 3.2. Correlation Between Body Composition and Site-Specific Bone Mineral Density

Pearson correlation analyses between demographic, anthropometric, and body composition variables and site-specific BMD are presented in Table 2.

Age and years since menopause were negatively correlated with BMD at the lumbar spine, total hip, and femoral neck (all *p* < 0.001). In contrast, body weight, height, BMI, WC, ASMI, total fat mass, percentage body fat, and trunk fat mass were positively correlated with BMD at all three skeletal sites (all *p* < 0.01).

Among the body composition measures, ASMI showed the strongest positive correlations with BMD across all three skeletal sites (lumbar spine: *r* = 0.424; total hip: *r* = 0.543; femoral neck: *r* = 0.498; all *p* < 0.001). WC was also positively correlated with BMD, with correlations of *r* = 0.211 at the lumbar spine, *r* = 0.384 at the total hip, and *r* = 0.311 at the femoral neck. Trunk fat mass showed similar positive correlations with BMD (*r* = 0.261, 0.357, and 0.332, respectively; all *p* < 0.001).

### 3.3. Multivariable Linear Regression Analysis

The results of the multivariable linear regression analyses are presented in Table 3. Absolute BMD (g/cm^2^) at the lumbar spine, total hip, and femoral neck was used as the dependent variable. Each model included age, years since menopause, WC, ASMI, HRT use, osteoporosis medication use, calcium supplementation, vitamin D supplementation, and fracture history. Robust standard errors were used to account for potential heteroskedasticity. No evidence of problematic multicollinearity was observed, with all variance inflation factors below 3.

After multivariable adjustment, ASMI was positively associated with BMD at all three skeletal sites. Each 1 kg/m^2^ higher ASMI was associated with a 0.077 g/cm^2^ higher lumbar spine BMD (95% CI, 0.045–0.108; standardized β = 0.362; *p* < 0.001), a 0.065 g/cm^2^ higher total hip BMD (95% CI, 0.043–0.087; standardized β = 0.371; *p* < 0.001), and a 0.056 g/cm^2^ higher femoral neck BMD (95% CI, 0.038–0.075; standardized β = 0.353; *p* < 0.001).

The association between WC and BMD varied by skeletal site. WC was positively associated with total hip BMD (unstandardized β = 0.0022 g/cm^2^ per cm; 95% CI, 0.0006–0.0038; standardized β = 0.158; *p* = 0.006), whereas no statistically significant associations were observed at the lumbar spine (β = 0.0001; 95% CI, −0.0021 to 0.0024; standardized β = 0.009; *p* = 0.899) or femoral neck (β = 0.0013; 95% CI, −0.0002 to 0.0029; standardized β = 0.105; *p* = 0.092).

Among the other covariates, osteoporosis medication use was inversely associated with BMD at all three skeletal sites. Age was inversely associated with femoral neck BMD (*p* = 0.014), but not with lumbar spine or total hip BMD. HRT use, calcium supplementation, vitamin D supplementation, and fracture history were not significantly associated with BMD at any of the three skeletal sites.

The adjusted R^2^ values were 0.266, 0.372, and 0.358 for the lumbar spine, total hip, and femoral neck models, respectively.

Supplementary analyses using site-specific T-scores as the outcomes showed a similar overall pattern, with ASMI remaining positively associated with T-scores at all three skeletal sites (Table S1).

### 3.4. Summary of Main Findings

Overall, ASMI showed consistent positive associations with BMD at the lumbar spine, total hip, and femoral neck after adjustment for demographic and available bone-related clinical factors. In contrast, the association between WC and BMD was site-specific, remaining statistically significant only at the total hip. These findings indicate that appendicular skeletal muscle mass is consistently associated with BMD across skeletal sites, whereas the relationship between central adiposity and BMD may vary according to anatomical site.

## 4. Discussion

### 4.1. Principal Findings

This real-world cross-sectional study investigated the associations of ASMI and abdominal adiposity, assessed by WC, with site-specific BMD in postmenopausal women. Three principal findings emerged. First, ASMI demonstrated consistent positive associations with BMD at the lumbar spine, total hip, and femoral neck after adjustment for age, years since menopause, and available bone-related clinical factors. Second, although WC was positively correlated with BMD at all skeletal sites in the unadjusted analyses, its association remained statistically significant only at the total hip after multivariable adjustment. Third, these findings suggest that skeletal muscle mass has a more consistent association with bone health than abdominal adiposity, whereas the relationship between central adiposity and BMD appears to be site-specific. Together, these results support the concept that different components of body composition may be differently associated with skeletal health and that reliance on body weight or BMI alone may not adequately reflect bone health in postmenopausal women.

### 4.2. Skeletal Muscle Mass and Site-Specific Bone Mineral Density

The most consistent finding of this study was the positive association between appendicular skeletal muscle mass and BMD across all skeletal sites. After adjustment for age, years since menopause, and available bone-related clinical factors, ASMI remained significantly associated with absolute BMD at the lumbar spine, total hip, and femoral neck. This observation is consistent with previous epidemiological studies demonstrating that appendicular muscle mass is associated with BMD, particularly among postmenopausal women and older adults [32,33,34,35].

The close relationship between skeletal muscle and bone is supported by the mechanostat theory, which proposes that bone continuously adapts to mechanical loading generated primarily by skeletal muscle contraction rather than by body weight alone [15]. In addition to mechanical loading, accumulating evidence indicates that skeletal muscle and bone function as an integrated endocrine unit through bidirectional signaling mediated by myokines, osteokines, and growth factors. Myokines released during muscle contraction, including insulin-like growth factor-1 (IGF-1), irisin, and other anabolic mediators, stimulate osteoblast differentiation and bone formation, whereas preservation of muscle mass contributes to maintaining skeletal integrity during aging [16,17,36].

Our findings further support the concept that appendicular muscle mass represents a clinically relevant marker of skeletal health in postmenopausal women. Unlike BMI, which does not distinguish lean tissue from adiposity, ASMI directly reflects appendicular skeletal muscle mass. This may explain why ASMI remained associated with BMD even after adjustment for abdominal adiposity and available bone-related clinical factors. Similar observations have been reported in population-based studies from the United States and Asia, where reduced muscle mass was consistently associated with lower BMD and an increased prevalence of osteopenia and osteoporosis [32,33,34,35].

From a clinical perspective, these findings suggest that preservation of skeletal muscle may be relevant to bone health in postmenopausal women. Assessment of muscle mass using bioelectrical impedance analysis may complement conventional anthropometric assessment by providing additional information on body composition. Because bioelectrical impedance analysis is relatively inexpensive, widely available, and easily implemented in outpatient settings, ASMI may represent a practical measure for characterizing musculoskeletal health in postmenopausal women [24,25,26,27]. However, the present study was not designed to evaluate the diagnostic or predictive performance of ASMI for osteoporosis or fracture risk.

### 4.3. Differential Associations of Abdominal Adiposity with Site-Specific Bone Mineral Density

In contrast to skeletal muscle mass, the association between abdominal adiposity and BMD was not consistent across skeletal sites. Although WC showed positive correlations with lumbar spine, total hip, and femoral neck BMD in the unadjusted analyses, only the association with total hip BMD remained statistically significant after multivariable adjustment. No statistically significant associations were observed at the lumbar spine or femoral neck. These findings suggest that the relationship between central adiposity and bone density may vary according to skeletal site.

The site-specific association observed for WC may reflect the complex interaction between the mechanical and metabolic effects of adiposity. Increased body mass may increase skeletal loading, particularly at weight-bearing regions of the hip, thereby contributing to higher BMD. However, visceral adipose tissue is also an active endocrine organ that secretes inflammatory cytokines, adipokines, and other bioactive mediators that may adversely influence bone remodeling. Consequently, the apparent positive association between obesity and bone density observed in unadjusted analyses may partly reflect mechanical loading rather than a direct beneficial effect of adipose tissue on bone metabolism [10,11,12,13,14,37,38,39,40]. In addition, degenerative changes and local artifacts at the lumbar spine may influence DXA-derived BMD measurements in older women and could partly contribute to the observed site-specific differences [4].

Our findings are consistent with previous studies reporting that body composition provides greater clinical information than BMI alone for evaluating skeletal health [8,9,41]. BMI reflects total body mass without distinguishing lean tissue from adipose tissue and therefore cannot differentiate the potentially different contributions of skeletal muscle and adipose tissue to bone health. Similarly, studies using imaging-based assessments of visceral adiposity have demonstrated that visceral fat may not confer uniform skeletal benefits and may even be associated with adverse changes in bone microarchitecture despite preserved areal BMD [19,20]. Together with the present findings, these observations support the concept that muscle and adipose tissue should be considered as biologically distinct components of body composition when assessing skeletal health.

These findings have practical implications for outpatient assessment. WC is inexpensive, non-invasive, and commonly used in routine metabolic risk assessment, whereas BIA enables rapid estimation of appendicular muscle mass during the same clinical visit. Combining these complementary measurements may provide a more comprehensive assessment of body composition than BMI alone. However, further studies are required to determine whether these measures provide additional value for osteoporosis risk prediction beyond established clinical assessment tools.

### 4.4. Clinical Implications

The present findings have several practical implications for the assessment of bone health in postmenopausal women. Current clinical assessment frequently includes body weight or BMI; however, these measures do not distinguish between lean tissue and adipose tissue. The present findings suggest that different components of body composition have distinct associations with regional bone density, indicating that BMI alone may not adequately characterize the relationship between body composition and skeletal health in this population.

Incorporating simple body composition assessment into routine outpatient practice may provide additional information regarding musculoskeletal health [23,24,25,26,27,28,29]. BIA provides a rapid, non-invasive, and relatively inexpensive estimate of appendicular skeletal muscle mass, whereas WC is already commonly used in cardiometabolic risk assessment. The combined assessment of ASMI and WC may therefore provide complementary information beyond conventional anthropometric measures without substantially increasing clinical workload.

These findings may be particularly relevant in outpatient settings where detailed body composition assessment is not routinely available. Although DXA remains the reference standard for the diagnosis of osteoporosis, assessment of muscle mass and abdominal adiposity may provide complementary information regarding musculoskeletal health. Further prospective studies are required to determine whether incorporating these measures into established osteoporosis assessment strategies improves clinical risk prediction or patient outcomes.

Nevertheless, the present findings should be interpreted within the context of an observational cross-sectional study and therefore should not be interpreted as evidence of causal relationships. Prospective longitudinal studies are required to determine whether changes in muscle mass or abdominal adiposity are associated with subsequent changes in BMD and fracture risk.

### 4.5. Strengths and Limitations

This study has several strengths. First, it was conducted in a real-world outpatient menopause clinic, reflecting routine clinical practice and enhancing the clinical applicability of the findings. Second, BMD was assessed using DXA, the current reference standard for osteoporosis diagnosis, while body composition was evaluated using multi-frequency BIA, a practical method that is readily applicable in routine outpatient settings. Third, the study simultaneously evaluated skeletal muscle mass and abdominal adiposity within the same multivariable models, allowing their associations with site-specific BMD to be examined after adjustment for demographic and available bone-related clinical factors.

The multivariable models included age, years since menopause, HRT use, osteoporosis medication use, calcium supplementation, vitamin D supplementation, and fracture history in addition to the primary body-composition variables. Nevertheless, several limitations should be acknowledged. First, the cross-sectional design precludes causal inference between body composition and BMD. Second, this was a single-center study conducted among postmenopausal women attending a specialized outpatient clinic, which may introduce selection bias and limit the generalizability of the findings to community-based populations, other clinical settings, and populations with different ethnic or demographic characteristics. Third, abdominal adiposity was assessed using WC rather than direct imaging techniques such as computed tomography or magnetic resonance imaging; therefore, visceral adipose tissue could not be quantified directly. Fourth, body composition was estimated using BIA rather than DXA-derived lean mass, although multi-frequency BIA has demonstrated acceptable agreement with DXA in previous validation studies [24,25,26]. Fifth, ASMI reflects muscle quantity and does not directly assess muscle strength, physical performance, or muscle quality. Finally, other potentially important factors related to bone health, including socioeconomic status, physical activity, nutritional status, serum vitamin D status, smoking, alcohol consumption, and relevant chronic diseases, were not available for inclusion in the multivariable models. Future prospective multicenter studies incorporating these factors, together with measures of muscle strength and physical performance and longitudinal fracture outcomes, are warranted to further clarify the relationships between muscle mass, adiposity, and skeletal health.

## 5. Conclusions

In this real-world cross-sectional study of postmenopausal women, appendicular skeletal muscle mass demonstrated consistent independent associations with BMD at the lumbar spine, total hip, and femoral neck, whereas the association between abdominal adiposity and BMD varied according to skeletal site. These findings suggest that skeletal muscle mass is more consistently associated with BMD than WC and support the concept that different components of body composition may have distinct relationships with skeletal health.

From a clinical perspective, assessment of appendicular skeletal muscle mass together with WC may provide complementary information on body composition and musculoskeletal health in routine outpatient practice. Future prospective longitudinal studies are needed to determine whether changes in skeletal muscle mass and central adiposity are associated with subsequent changes in BMD and fracture risk in postmenopausal women.

## Figures and Tables

**Table 1:** Baseline characteristics of the study participants according to appendicular skeletal muscle mass index (ASMI) status.

Characteristic	Low ASMI (n = 177)	Normal ASMI (n = 168)	*p*-Value
**Demographic characteristics**			
Age, years	61.3 ± 8.4	59.9 ± 8.0	0.111
Years since menopause	12.4 ± 7.5	10.9 ± 6.9	0.074
**Anthropometric measurements**			
Body weight, kg	49.9 ± 6.1	63.9 ± 8.3	<0.001
Height, cm	153.8 ± 5.2	156.5 ± 5.0	<0.001
BMI, kg/m^2^	21.1 ± 2.7	26.2 ± 3.5	<0.001
Waist circumference, cm	74.6 ± 7.9	85.1 ± 8.1	<0.001
**Body composition**			
ASMI, kg/m^2^	5.14 ± 0.40	6.34 ± 0.50	--
Total fat mass, kg	17.4 ± 4.7	24.8 ± 6.4	<0.001
Body fat, %	34.3 ± 5.9	38.2 ± 5.4	<0.001
Trunk fat mass, kg	9.5 ± 2.9	13.5 ± 3.5	<0.001
**Bone mineral density**			
Lumbar spine BMD, g/cm^2^	0.923 ± 0.142	1.030 ± 0.162	<0.001
Total hip BMD, g/cm^2^	0.788 ± 0.110	0.915 ± 0.123	<0.001
Femoral neck BMD, g/cm^2^	0.729 ± 0.105	0.829 ± 0.113	<0.001
Lumbar spine T-score	−1.53 ± 1.21	−0.67 ± 1.36	<0.001
Total hip T-score	−1.29 ± 0.93	−0.27 ± 1.00	<0.001
Femoral neck T-score	−1.45 ± 1.03	−0.68 ± 0.99	<0.001
**Bone-related clinical characteristics**			
HRT use, n/N (%)	10/167 (6.0)	12/155 (7.7)	0.533
Osteoporosis medication use, n/N (%)	35/167 (21.0)	10/155 (6.5)	<0.001
Calcium supplementation, n/N (%)	144/173 (83.2)	121/161 (75.2)	0.068
Vitamin D supplementation, n/N (%)	128/172 (74.4)	107/161 (66.5)	0.111
Fracture history, n/N (%)	8/169 (4.7)	8/155 (5.2)	0.859
**Bone status, n (%)**			
Normal	26 (14.7%)	76 (45.2%)	<0.001
Osteopenia	93 (52.5%)	79 (47.0%)	
Osteoporosis	58 (32.8%)	13 (7.7%)	

Data are presented as mean ± standard deviation (SD) unless otherwise indicated. For categorical variables with missing data, values are presented as n/N (%), where N represents the number of participants with available data. The number of observations varied for some continuous and categorical variables because of missing data. Between-group comparisons were performed using the independent-samples *t* test or Mann–Whitney *U* test for continuous variables, as appropriate, and the chi-square test or Fisher’s exact test for categorical variables. No statistical test was performed for ASMI because ASMI was used to define the study groups. ASMI, appendicular skeletal muscle mass index; BMD, bone mineral density; BMI, body mass index; HRT, hormone replacement therapy; WC, waist circumference.

**Table 2:** Pearson correlation coefficients between demographic, anthropometric, and body composition variables and site-specific bone mineral density.

Variable	Lumbar Spine BMD (g/cm^2^)	Total Hip BMD (g/cm^2^)	Femoral Neck BMD (g/cm^2^)
**Demographic characteristics**			
Age, years	−0.268 **	−0.295 **	−0.367 **
Years since menopause	−0.261 **	−0.279 **	−0.349 **
**Anthropometric measurements**			
Body Weight, kg	0.374 **	0.488 **	0.466 **
Height, cm	0.213 **	0.152 **	0.266 **
BMI, kg/m^2^	0.310 **	0.453 **	0.382 **
Waist Circumference, cm	0.211 **	0.384 **	0.311 **
**Body composition**			
ASMI, kg/m^2^	0.424 **	0.543 **	0.498 **
Total Fat Mass, kg	0.284 **	0.380 **	0.362 **
Body Fat, %	0.163 **	0.213 **	0.197 **
Trunk fat mass, kg	0.261 **	0.357 **	0.332 **

Data are presented as Pearson correlation coefficients (*r*). Pairwise complete observations were used; therefore, the number of observations varied across analyses because of missing data. ** *p* < 0.01. ASMI, appendicular skeletal muscle mass index; BMD, bone mineral density; BMI, body mass index; WC, waist circumference.

**Table 3:** Multivariable linear regression analyses of factors associated with site-specific bone mineral density.

Predictor	Unstandardized β	95% CI for β	Standardized β	*p*-Value
**Lumbar spine BMD, g/cm^2^ (n = 299; adjusted R^2^ = 0.266)**				
Age, years	−0.0001	−0.0036 to 0.0034	−0.005	0.959
Years since menopause	−0.0027	−0.0064 to 0.0011	−0.122	0.159
Waist circumference, cm	0.0001	−0.0021 to 0.0024	0.009	0.899
ASMI, kg/m^2^	0.0766	0.0454 to 0.1077	0.362	<0.001
HRT use	0.0110	−0.0410 to 0.0630	0.017	0.677
Osteoporosis medication use	−0.0977	−0.1418 to −0.0535	−0.219	<0.001
Calcium supplementation	0.0147	−0.0387 to 0.0680	0.038	0.589
Vitamin D supplementation	−0.0374	−0.0810 to 0.0063	−0.108	0.093
Fracture history	0.0565	−0.0312 to 0.1442	0.080	0.206
**Total hip BMD, g/cm^2^ (n = 298; adjusted R^2^ = 0.372)**				
Age, years	−0.0016	−0.0040 to 0.0009	−0.094	0.211
Years since menopause	−0.0015	−0.0043 to 0.0012	−0.085	0.276
Waist circumference, cm	0.0022	0.0006 to 0.0038	0.158	0.006
ASMI, kg/m^2^	0.0650	0.0435 to 0.0866	0.371	<0.001
HRT use	0.0263	−0.0297 to 0.0823	0.049	0.356
Osteoporosis medication use	−0.0536	−0.0894 to −0.0179	−0.145	0.003
Calcium supplementation	−0.0232	−0.0681 to 0.0217	−0.072	0.310
Vitamin D supplementation	−0.0019	−0.0418 to 0.0380	−0.007	0.926
Fracture history	0.0505	−0.0058 to 0.1068	0.087	0.079
**Femoral neck BMD, g/cm^2^ (n = 299; adjusted R^2^ = 0.358)**				
Age, years	−0.0029	−0.0052 to −0.0006	−0.193	0.014
Years since menopause	−0.0017	−0.0043 to 0.0009	−0.104	0.194
Waist circumference, cm	0.0013	−0.0002 to 0.0029	0.105	0.092
ASMI, kg/m^2^	0.0563	0.0375 to 0.0750	0.353	<0.001
HRT use	0.0327	−0.0107 to 0.0762	0.067	0.139
Osteoporosis medication use	−0.0330	−0.0641 to −0.0020	−0.098	0.037
Calcium supplementation	−0.0102	−0.0537 to 0.0334	−0.035	0.646
Vitamin D supplementation	−0.0027	−0.0410 to 0.0356	−0.010	0.891
Fracture history	0.0500	−0.0074 to 0.1075	0.094	0.088

BMD was expressed in g/cm^2^. Models included age, years since menopause, waist circumference, ASMI, HRT use, osteoporosis medication use, calcium supplementation, vitamin D supplementation, and fracture history. Robust standard errors were used. Regression results are presented as unstandardized regression coefficients (β), standardized regression coefficients (standardized β), robust 95% confidence intervals (CIs), and corresponding *p* values. Analyses were based on complete cases for the variables included in each model. ASMI, appendicular skeletal muscle mass index; BMD, bone mineral density; CI, confidence interval; HRT, hormone replacement therapy.

## Data Availability

The datasets generated and analyzed during the current study are available from the corresponding author upon reasonable request. The data are not publicly available because they contain information that could compromise the privacy of research participants and are subject to institutional ethical restrictions.

## Supplementary Materials

The following supporting information can be downloaded at: https://www.mdpi.com/article/10.3390/healthcare14162663/s1, Table S1: Multivariable linear regression analyses of factors associated with site-specific bone mineral density T-scores.

## Abbreviations

**Abbreviation**	**Definition**
ASMI	Appendicular Skeletal Muscle Mass Index
AWGS	Asian Working Group for Sarcopenia
BIA	Bioelectrical Impedance Analysis
BMD	Bone Mineral Density
BMI	Body Mass Index
CI	Confidence Interval
DXA	Dual-energy X-ray Absorptiometry
IL-6	Interleukin-6
IRB	Institutional Review Board
OPG	Osteoprotegerin
RANKL	Receptor Activator of Nuclear Factor Kappa-B Ligand
SD	Standard Deviation
STROBE	Strengthening the Reporting of Observational Studies in Epidemiology
SUTH	Suranaree University of Technology Hospital
TNF-α	Tumor Necrosis Factor-alpha
VIF	Variance Inflation Factor
WC	Waist Circumference
WHO	World Health Organization
