# Supplementary material for: Independent Associations of Appendicular Skeletal Muscle Mass Index and Waist Circumference with Site-Specific Bone Mineral Density in Postmenopausal Women: A Real-World Cross-Sectional Study
Source: Healthcare (Basel). 2026 Aug 21;14(16):2663. doi: 10.3390/healthcare14162663 (PMC13511964; doi:10.3390/healthcare14162663)
Supplement: Supplementary file 1 [file healthcare-14-02663-s001.zip › healthcare-4453069-supplementary.pdf]

Supplementary Materials

Supplementary Table S1. Multivariable linear regression analyses of factors associated with site-specific bone mineral density T-scores.

Predictor	Unstandardized β	95% CI for β	Standardized β	*p*-value
**Lumbar spine T-score (n = 299; adjusted R^2 = 0.264)**				
Age, years	0.0026	−0.0266 to 0.0317	0.015	0.861
Years since menopause	−0.0244	−0.0552 to 0.0063	−0.132	0.119
Waist circumference, cm	−0.0001	−0.0189 to 0.0187	−0.001	0.992
ASMI, kg/m²	0.6536	0.3911 to 0.9160	0.367	< 0.001
HRT use	0.1589	−0.2849 to 0.6027	0.029	0.482
Osteoporosis medication use	−0.8534	−1.2235 to −0.4833	−0.226	< 0.001
Calcium supplementation	0.1545	−0.2912 to 0.6001	0.047	0.496
Vitamin D supplementation	−0.3133	−0.6812 to 0.0546	−0.107	0.095
Fracture history	0.4456	−0.2875 to 1.1787	0.075	0.233
**Total hip T-score (n = 298; adjusted R^2 = 0.326)**				
Age, years	−0.0028	−0.0243 to 0.0187	−0.020	0.799
Years since menopause	−0.0181	−0.0423 to 0.0061	−0.121	0.142
Waist circumference, cm	0.0204	0.0066 to 0.0343	0.179	0.004
ASMI, kg/m²	0.4846	0.2921 to 0.6771	0.336	< 0.001
HRT use	0.4120	−0.0904 to 0.9144	0.093	0.108
Osteoporosis medication use	−0.3166	−0.6288 to −0.0044	−0.104	0.047
Calcium supplementation	−0.1797	−0.6096 to 0.2502	−0.067	0.411
Vitamin D supplementation	−0.0696	−0.4453 to 0.3061	−0.029	0.716
Fracture history	0.4129	−0.0140 to 0.8399	0.086	0.058
**Femoral neck T-score (n = 299; adjusted R^2 = 0.267)**				
Age, years	−0.0124	−0.0355 to 0.0107	-0.091	0.290
Years since menopause	−0.0196	−0.0450 to 0.0058	-0.132	0.129
Waist circumference, cm	0.0149	−0.00003 to 0.0298	0.131	0.050
ASMI, kg/m²	0.3960	0.2111 to 0.5808	0.275	< 0.001
HRT use	0.2933	−0.0660 to 0.6526	0.066	0.109
Osteoporosis medication use	−0.3899	−0.6753 to −0.1044	−0.128	0.008
Calcium supplementation	−0.1989	−0.6078 to 0.2101	−0.075	0.339
Vitamin D supplementation	0.0498	−0.2777 to 0.3773	0.021	0.765
Fracture history	0.4504	−0.1033 to 1.0041	0.094	0.110

Data are presented as unstandardized regression coefficients, standardized regression coefficients, robust 95% confidence intervals (CIs), and corresponding *p* values. Models included age, years since menopause, waist circumference, ASMI, HRT use, osteoporosis medication use, calcium supplementation, vitamin D supplementation, and fracture history. Heteroskedasticity-robust standard errors were used. Analyses were based on complete cases for the variables included in each model. ASMI, appendicular skeletal muscle mass index; BMD, bone mineral density; CI, confidence interval; HRT, hormone replacement therapy.
